# Effect of Theobromine Consumption on Serum Lipoprotein Profiles in Apparently Healthy Humans with Low HDL-Cholesterol Concentrations

**DOI:** 10.3389/fmolb.2017.00059

**Published:** 2017-08-24

**Authors:** Doris M. Jacobs, Lotte Smolders, Yuguang Lin, Niels de Roo, Elke A. Trautwein, John van Duynhoven, Ronald P. Mensink, Jogchum Plat, Velitchka V. Mihaleva

**Affiliations:** ^1^Unilever R&D Vlaardingen, Netherlands; ^2^Department of Human Biology, School of Nutrition and Translational Research in Metabolism, Maastricht University Maastricht, Netherlands; ^3^Laboratory of Biophysics, Wageningen University Wageningen, Netherlands

**Keywords:** theobromine, NMR, lipoprotein, HDL, PLS model

## Abstract

**Scope:** Theobromine is a major active compound in cocoa with allegedly beneficial effect on high-density-lipoprotein-cholesterol (HDL-CH). We have investigated the effect of theobromine (TB) consumption on the concentrations of triglyceride (TG) and cholesterol (CH) in various lipoprotein (LP) subclasses.

**Methods:** In a randomized, double-blind, placebo-controlled, cross-over study, 44 apparently healthy women and men (age: 60 ± 6 years, BMI: 29 ± 3 kg/m^2^) with low baseline HDL-CH concentrations consumed a drink supplemented with 500 mg/d theobromine for 4 weeks. TG and CH concentrations in 15 LP subclasses were predicted from diffusion-edited ^1^H NMR spectra of fasting serum.

**Results:** The LP phenotype of the subjects was characterized by low CH concentrations in the large HDL particles and high TG concentrations in large VLDL and chylomicron (CM) particles, which clearly differed from a LP phenotype of subjects with normal HDL-CH. TB only reduced CH concentrations in the LDL particles by 3.64 and 6.79%, but had no effect on TG and CH in any of the HDL, VLDL and CM subclasses.

**Conclusion:** TB was not effective on HDL-CH in subjects with a LP phenotype characterized by low HDL-CH and high TG in VLDL.

## Introduction

Theobromine (TB) is postulated as one of the active constituents explaining the cardioprotective effects of cocoa products, dark chocolate and tea (Berends et al., 2015). Amongst others, TB has been shown to lower blood pressure in healthy (Mitchell et al., 2011) and hypertensive (van den Bogaard et al., 2010) subjects. Moreover, in healthy subjects consuming 850 mg theobromine for 4 weeks, serum high-density-lipoprotein-cholesterol (HDL-CH) and apolipoprotein A-I (apoA-I) concentrations increased by 10 and 8%, respectively (Neufingerl et al., 2013).

Plasma HDL-CH concentrations have consistently been shown to be inversely associated with coronary heart disease (CHD) risk in large-scale epidemiological studies (Boden, 2000). However, a number of more recent intervention studies with drugs such as Niacin, cholesteryl ester transfer protein (CETP) inhibitors and Fibrates (added to statin treatment versus statin alone) have shown that increasing plasma HDL-CH and apoA-I did not result in consistent cardio-protective effects as reviewed by Choi et al. (2017). This has led to the current paradigm that raising HDL-CH concentrations as such is not a target for CHD risk lowering. HDL particles are heterogeneous in lipid and apoA-I contents as well as in particle size, which may reflect metabolic status and functionality of HDL. For example, pre-beta HDL and the smaller HDL (HDL-3) particles seem to interact with the ATP-binding cassette transporter A1 (ABCA1) on macrophages facilitating cholesterol ester (CE) efflux, whereas the larger HDL subclass (HDL-2) is the acceptor for ABCG1 (Wang et al., 2004; Hafiane et al., 2015).

Theobromine is thought to have cardio-protective properties by the following mechanism. Theobromine has been shown to inhibit phosphodiesterases (Sugimoto et al., 2014). Phosphodiesterases can degrade cAMP (Essayan, 2001). Cellular cAMP increases ABCA1 activity (Lee et al., 2002), which plays a role in CH efflux from macrophage foam cells to apoA-I, the principal apolipoprotein of HDL. In this way, theobromine may increase HDL-CH and exert the cardio-protective effect. Thus, it is relevant to evaluate changes in the composition and sizes of HDL subclasses upon theobromine to be able to elucidate the mechanism behind the relationship between HDL particles and CHD risk.

Nuclear magnetic resonance (NMR) spectroscopy is well known for its suitability to classify lipoproteins (LP) into a number of LP subclasses including various HDL, LDL, and VLDL subclasses. This comprehensive LP profile has been considered as more accurate estimates of CHD risk as compared to the total VLDL, LDL, and HDL classification (Kuller et al., 2002; Mallol et al., 2013). For instance, changes in small dense LDL particles and some HDL subclasses have particularly been associated with CHD risk (Lamarche et al., 1997; Pirillo et al., 2013). Furthermore, in a recent study we have observed significant changes on HDL subclasses upon a dietary treatment despite unaltered total HDL concentrations (Jacobs et al., 2015). In this context, investigating triglycerides (TG) and CH contents in HDL subclasses and other LP subclasses is of scientific interest, since it may help to understand how changes in LP compositions may be linked to LP functionality.

Recently, we have assessed the effect of 4 weeks TB consumption on serum lipid and total LP concentrations in subjects with low serum HDL-CH concentrations at baseline (Smolders et al., 2017). We observed an increased trend in fasting total HDL-CH concentrations and a significant reduction in total LDL-CH concentrations. In the present study, we have investigated the effect of TB on various LP subclasses in more depth using the serum samples from this study. For this purpose, the TG and CH concentrations in different LP subclasses were predicted from diffusion-edited ^1^H NMR spectra using Partial Least Squares (PLS) modeling. The LP distributions from subjects with low baseline HDL-CH measured in this study were compared with those from a previously reported study with hypercholesterolemic subjects (Mihaleva et al., 2014) to understand differences in LP phenotypes between these two study cohorts. Furthermore, the effects of TB on the TG and CH composition in HDL and other LP subclasses are described.

## Materials and methods

### Study design

The complete study design has been reported in detail earlier (Smolders et al., 2017). In brief, the study was designed as a randomized, double-blind, placebo-controlled, cross-over study. The study was performed according to the guidelines laid down in the Declaration of Helsinki with written informed consent from all subjects. The study was approved by the Medical Ethical Committee of Maastricht University and registered at clinicaltrials.gov (NCT02209025). In total, 30 healthy men and 18 healthy women (age: 60 ± 6 years, BMI: 29 ± 3 kg/m^2^) were recruited. Four subjects dropped out of the study. At baseline, subjects had fasting serum HDL-CH concentrations <1.2 mmol/L for men and <1.5 mmol/L for women, which were below the 50th percentile of serum HDL-CH concentrations in the Dutch population (Bos et al., 2007). During the two 4-week treatment periods, subjects consumed daily either an instant drink supplemented with 500 mg TB (Fragon, Uitgeest, The Netherlands) or the same drink without added TB (placebo) with breakfast. The two cross-over treatment periods were separated by a 4-week wash-out period, during which the subjects did not consume any of these drinks. Over the whole period, subjects were asked to refrain from consuming TB containing products (such as chocolate), to limit the consumption of caffeine containing drinks (such as coffee, tea, cola) to maximal four drinks a day and to follow their usual diet and physical activity.

### Blood sampling

Venous blood was collected after an overnight fast from each subject at the start of both intervention periods (D1 and D57) and twice in week 4 (D25, D28, D81, and D84) of both treatment periods. Serum samples for the NMR LP analysis were prepared by centrifuging the blood samples at 2,400 × g for 5 min. The separated serum samples were aliquoted to 0.5 mL/tube and stored at −80°C until analysis after the study was completed.

### NMR data acquisition

In total, 263 serum samples were used for NMR analysis. One sample (Subject 8, Day 81 placebo treatment) was missing. Frozen serum samples were slowly warmed up to room temperature and gently homogenized. Next, the samples were centrifuged at 600 × g for 30 s at room temperature. Equal volumes of 100 μL of serum sample and phosphate buffer (prepared of 0.075 M Na_2_HPO_4_x7H_2_O adjusted to pH 7.4, 5.5 mM sodium 3-trimethylsilyl[2,2,3,3-*d*_4_] propionate, 0.12 M NaN_3_ and 10% D_2_O) were transferred to an Eppendorf tube. The NMR samples were centrifuged at 3,000 × g for 2 min. The mixture was transferred to a 3-mm SampleJet rack tube and measured at 310 K (calibrated temperature) on a Bruker Avance III NMR spectrometer, operating at 600.25 MHz and equipped with a 5-mm cryoprobe and a gradient unit with a maximum gradient strength of 53 G/cm. Each sample was equilibrated at 310 K for 5 min before data acquisition. ^1^H NMR NOESY (NOESYGPPR1D) spectra and diffusion-edited (LEDBPGPPR2S1D) spectra with a gradient strength of 80% were acquired on each sample using the same acquisition parameters. A spectral window of 30 ppm, an acquisition time of 2.7 s, a mixing time of 10 ms and a water pre-saturation time of 4 s were used. For each sample 32 scans were accumulated. Each dataset was automatically processed using a line broadening of 1 Hz. The NOESY data were automatically aligned to the left signal of the alanine doublet at 1.49 ppm. Each diffusion-edited spectrum was aligned to its corresponding NOESY spectrum by using its settings for the offset and spectral frequency.

### Quantification of CH and TG concentrations in LP subclasses

CH and TG concentrations in LP subclasses were predicted from the diffusion-edited ^1^H NMR spectra. Initially, these concentrations were predicted using the PLS model that has previously been established for a healthy, hypercholesterolemic cohort (henceforth called Mihaleva model; Mihaleva et al., 2014). Moreover, an additional PLS model was built on a subset of 122 samples from the current study (henceforth called TB model). This model was then used to predict the TG and CH concentrations in the LP subclasses of the remaining 141 samples.

The 122 samples were selected using principal component analysis (PCA) based on the methyl signals between 0.6 and 1.04 ppm. They were selected such that they were equally distributed across the largest variation. These samples were from 31 subjects at baseline and from 30 subjects after the 4-week intake of either placebo or TB. Details on how the PLS model was built and validated have previously been described (Mihaleva et al., 2014). In brief, a second aliquot of the selected samples was analyzed by LipoSEARCH (Skylight Biotech Inc., Akita, Japan) using high performance liquid chromatography (HPLC). This analysis reported the CH and TG concentrations in four main classes (chylomicrons (CM), VLDL, LDL, and HDL) and 20 LP subclasses in mg/dL. The concentrations in mg/dL were converted to mmol/L using molecular masses of 385 and 884 g/mol for CH and TG, respectively (Okazaki et al., 2005).

The Kennard-Stone method (Kennard and Stone, 1969) was used to split the 122 samples into a training set of 80 samples and a test set of 42 samples. The training set comprised of 20 samples from D1, D28, D57, D84, respectively. The test set included 11 samples from D1 and D28 and 10 samples from D57 and D84, respectively. First, the PLS models were built on the training set. The X matrix consisted of 1746 data points for the TG models representing the lipid methylene and methyl signals between 1.4 and 0.6 ppm and of 961 data points for the CH model representing only the methyl signal between 1.04 and 0.6 ppm (961 data points). The Y matrix consisted of the TG or CH concentrations of a single LP subclass. If the distributions of the TG and CH concentrations were not symmetric, square root transformation was applied. Both the X and Y matrices were mean-centered. In total, 48 PLS models were calculated. Usually, 5-12 latent variables were selected using cross-validation. The significance of each training model was determined by permutation tests of the Y variable using a 10-fold cross-validation with the same number of latent variables as the model with the actual Y variable. The permutation tests were repeated 1,000 times. Statistically significant models (*p* < 0.001) were obtained for the TG and CH concentrations in total VLDL (particle size: 30–80 nm), total LDL (particle size: 16–30 nm), total HDL (particle size: 8–16 nm) and in 15 LP subclasses, including 2 chylomicrons (particle sizes: CM1: > 90 nm, CM2: 78 nm), 5 VLDLs (particle sizes: VLDL03: 64.0 nm, VLDL04: 53.6 nm, VLDL05: 44.5 nm, VLDL06: 36.8 nm, VLDL07: 31.3 nm), 4 LDLs (particle sizes: LDL08: 28.6 nm, LDL09: 25.5 nm, LDL10: 23.0 nm, LDL11: 20.7 nm,) and 4 HDLs (particle sizes: HDL15: 13.5 nm, HDL16: 12.1 nm, HDL17: 10.9 nm, HDL18: 9.8 nm). These models were then tested with the independent test set. The quality of the predictions from both the training and test sets was expressed in Q^2^, the root-mean-square error (RMSE) and the coefficient of variance (CV) as defined in Mihaleva et al. (2014). Moreover, the Pearson correlation coefficient (R) between the predicted and measured TG/CH concentrations was determined for each model. All calculations were performed using Matlab R2015a (The MathWorks).

### Statistical analysis of treatment effect

The effect of TB was assessed on the predicted TG and CH concentrations in 15 LP subclasses and in total LPs. The data set included 263 LP profiles from 44 subjects and from the pre- (D1 and D57) and post- (D25, D28, D81, D84) intervention periods. PCA was performed on mean-centered and unit-variance scaled data to detect outliers, trends, patterns and groupings. The analysis was performed in SIMCA P + version 12 software (Umetrics, Umea, Sweden). PCA revealed outliers from all six samples of three subjects (S4, S39, S47). These data were excluded from further analysis.

Analysis of variance (ANOVA) was performed using JMP® Pro 12.0.1 (SAS Institute Inc. 2013, Cary, North Carolina, U.S.A.) A mixed-effects ANOVA model was built using the fixed factors treatment (placebo, TB), baseline (D1, D57), gender, age, BMI, and repeated visits (1 for D25/D81; 2 for D28/D84). Subjects were set as random factor. The model was applied on non-transformed data, because the TG and CH concentrations were normally distributed. The adequacy of the model structure was assessed by visually inspecting the residuals in the scatter plots of standardized residuals versus fitted values. Post-hoc multiple comparisons of the LSMeans using a Dunnett-Hsu adjustment were performed, in case significant differences were detected by the linear mixed model. A value of *p* < 0.05 was considered statistically significant.

## Results

### PLS model

Initially, PLS models were built on a training set of 80 serum samples, which were subsequently tested on an independent set of 42 serum samples. These models were able to predict the TG and CH concentrations in LP subclasses—as measured by the HPLC method from LipoSEARCH—from diffusion-edited ^1^H NMR spectra. For the TG models both the methylene and methyl signals were used, while the CH models took only the methyl signals into account. Statistical significant models (*p* < 0.001) were obtained for TG and CH of 15 out of 20 LP subclasses and of the total VLDL, LDL, and HDL classes. The models for the smallest LDL (LDL12, LDL13), the largest HDL (HDL14) and the smallest HDL (HDL19, HDL20) subclasses were statistically not significant because of the low concentrations and the narrow concentration ranges of TG and CH in these particles. The performance of the statistically significant models are summarized in Tables 1, 2. High Q^2^, low RMSE and low CV values indicate good models. Plots showing the predicted versus measured TG and CH concentrations of the training and test sets are shown in Figure S1. Overall, the predicted values were comparable to the measured values. The performance parameters of the training and test sets were in good agreement, meaning that the model can be used for independent samples as long as the TG and CH concentrations of the independent sample are within the concentration range of the models. Most models were strong, with Q^2^ values larger than 0.6. Weaker models were obtained for VLDL07-CH (Q^2^ = 0.42), LDL08-CH (Q^2^ = 0.55), and HDL16-TG (Q^2^ = 0.50). The models for HDL15-CH (Q^2^ = 0.19) and HDL15-TG (Q^2^ = 0.11) were the weakest, yet statistically significant, due to the low concentrations and the narrow ranges of TG and CH in these particles. The predicted TG/CH concentrations strongly correlated with the measured values for all statistically significant models. The lowest correlation coefficient was 0.72. The errors in prediction (RMSE) were lower than 5 mg/dL, except for VLDL_tot_-TG, VLDL_tot_-CH, and LDL_tot_-CH. The coefficients of variations (CV) were in most cases lower than 15%.

**Table 1:** Summary of the statistically significant (*p* < 0.001) PLS models of cholesterol (CH) concentrations showing the number of latent variables (#LV), Q^2^, the correlation coefficient (R), the root-mean-square error (RMSE), and the coefficient of variation (CV) for the test and training sets, respectively.

**LP**	**#LV**	**Test**				**Training**^**a**^			
		**Q^2^**	**R**	**RMSE^b^**	**%CV**	**Q^2^**	**R**	**RMSE^b^**	**%CV**
CM01^c^	5	0.72	0.86	1.28	37.66	0.71	0.85	1.52	44.56
CM02^c^	9	0.81	0.91	0.47	23.97	0.81	0.90	0.64	30.39
VLDL03^c^	6	0.82	0.91	0.97	17.12	0.80	0.90	1.38	22.39
VLDL04	5	0.89	0.95	1.05	13.59	0.86	0.93	1.55	17.78
VLDL05	6	0.76	0.88	2.57	12.51	0.79	0.89	3.24	14.35
VLDL06	11	0.61	0.84	1.91	19.50	0.74	0.86	2.11	18.59
VLDL07	10	0.42	0.75	1.41	18.77	0.75	0.87	1.34	15.95
LDL08	11	0.55	0.80	3.82	12.57	0.76	0.87	4.31	14.19
LDL09	10	0.78	0.89	4.25	7.97	0.91	0.95	4.53	8.55
LDL10	10	0.68	0.84	3.28	15.85	0.84	0.92	3.43	15.75
LDL11	11	0.61	0.80	0.98	16.74	0.78	0.89	1.06	17.41
HDL15	11	0.19	0.59	0.16	13.24	0.51	0.72	0.15	11.85
HDL16^c^	12	0.90	0.95	0.57	16.67	0.78	0.89	1.18	27.07
HDL17	12	0.77	0.88	1.43	10.39	0.81	0.90	1.53	10.12
HDL18^c^	5	0.86	0.93	0.60	3.84	0.85	0.92	0.75	4.70
VLDL_tot_	10	0.84	0.93	5.26	10.27	0.86	0.93	6.77	11.82
LDL_tot_	11	0.88	0.94	6.51	5.84	0.92	0.96	8.58	7.62
HDL_tot_	12	0.86	0.93	2.06	5.19	0.87	0.94	2.51	5.88

a *Parameters calculated from 10-fold cross-validation*.

b *RMSE in mg/dL*.

c *Concentrations were square root transformed*.

**Table 2:** Summary of the statistically significant (*p* < 0.001) PLS models of triglyceride (TG) concentrations showing the number of latent variables (#LV), Q^2^, the correlation coefficient (R), the root-mean-square error (RMSE), and the coefficient of variation (CV) for the test and training sets, respectively.

**Lipid**	**#LV**	**Test**				**Training**^**a**^			
		**Q^2^**	**R**	**RMSE^b^**	**%CV**	**Q^2^**	**R**	**RMSE^b^**	**%CV**
CM01^c^	5	0.82	0.92	4.74	32.04	0.82	0.91	4.98	36.77
CM02^c^	6	0.92	0.96	1.40	15.23	0.90	0.96	1.87	20.72
VLDL03^c^	6	0.89	0.94	2.89	13.56	0.91	0.96	3.36	15.22
VLDL04	8	0.86	0.94	4.03	13.31	0.93	0.97	3.65	11.39
VLDL05	7	0.81	0.90	3.96	13.20	0.87	0.94	4.16	12.85
VLDL06	6	0.75	0.87	2.01	16.19	0.80	0.89	2.25	16.44
VLDL07	6	0.87	0.93	0.56	10.41	0.86	0.93	0.75	12.49
LDL08^c^	9	0.87	0.95	0.76	8.43	0.90	0.95	0.92	9.40
LDL09	7	0.86	0.94	0.82	9.10	0.93	0.96	0.76	7.92
LDL10^c^	7	0.81	0.91	0.46	13.02	0.86	0.93	0.48	12.71
LDL11^c^	7	0.81	0.91	0.16	13.48	0.86	0.93	0.17	13.24
HDL15^c^	11	0.11	0.38	0.27	56.31	0.63	0.80	0.12	24.47
HDL16^c^	11	0.50	0.71	0.52	34.30	0.78	0.88	0.55	29.05
HDL17^c^	8	0.76	0.91	0.58	13.00	0.72	0.86	0.83	16.86
HDL18^c^	8	0.64	0.82	0.61	16.04	0.55	0.75	0.80	20.10
VLDL_tot_	7	0.89	0.95	10.65	10.72	0.94	0.97	9.74	9.18
LDL_tot_	7	0.90	0.96	1.80	7.69	0.94	0.97	1.76	7.00
HDL_tot_	9	0.73	0.86	1.63	13.03	0.75	0.87	2.05	14.98

a *Parameters calculated from 10-fold cross-validation*.

b *RMSE in mg/dL*.

c *Concentrations were square root transformed*.

### Comparison of different LP phenotypes

**Figure 1:**
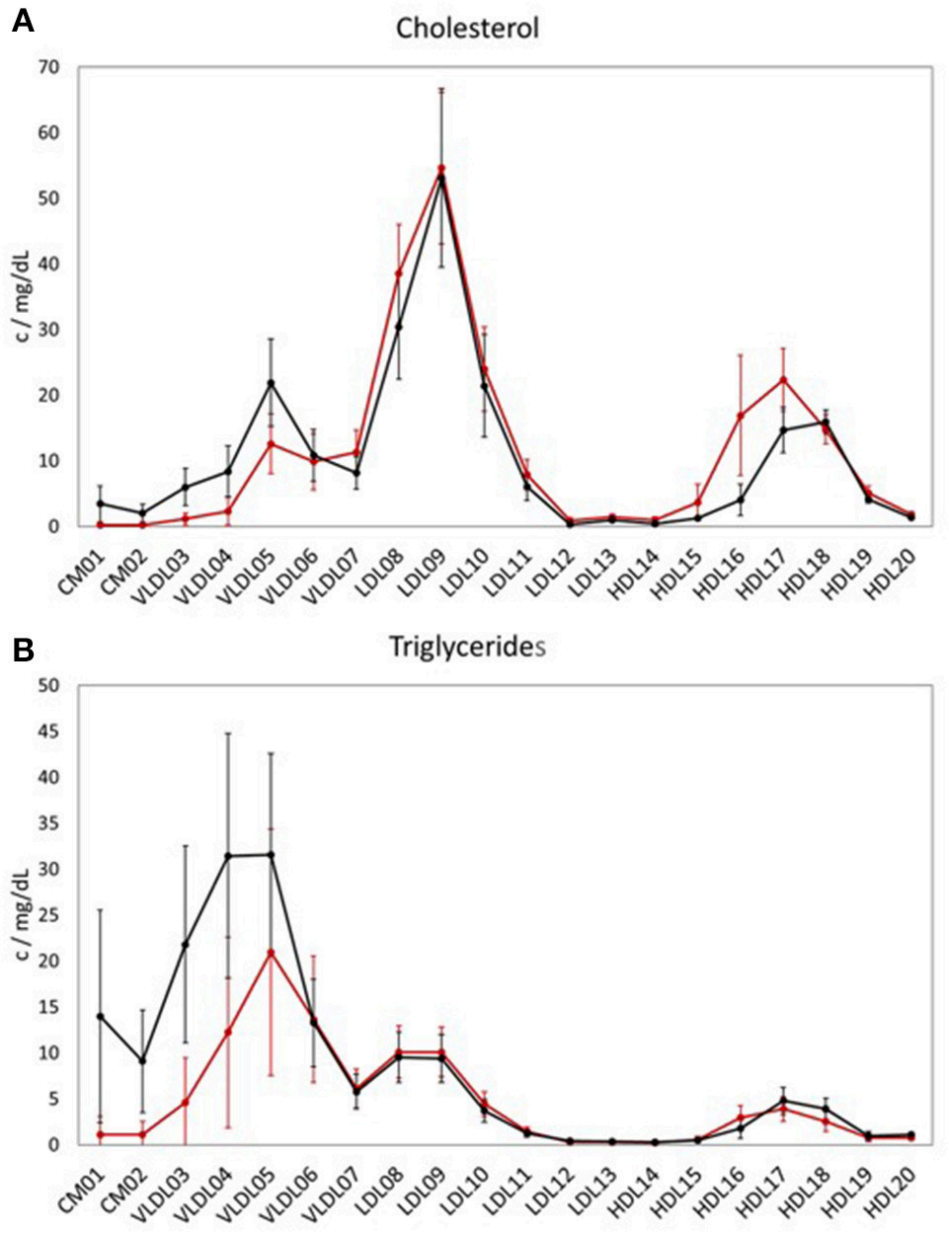
Lipoprotein profiles of two study cohorts. Mean concentrations of cholesterol (CH) **(A)** and triglycerides (TG) **(B)** in the LP subclasses in 122 subjects from the TB cohort (black curve) and in 290 subjects from the Mihaleva cohort (red curve). The concentrations were measured in mg/dL by LipoSEARCH. The error bars indicate standard deviations.

When comparing the LP distribution (based on the data from LipoSEARCH) from the TB cohort of this study with the healthy, hypercholesterolemic Mihaleva cohort from our previous study (Figure 1; Mihaleva et al., 2014), we observed lower HDL-CH in the larger HDL particles (HDL15, HDL16, HDL17), yet not in the smaller HDL particles (HDL18, HDL19, HDL20). At the same time, the TG concentrations were lower in the HDL16 and higher in the HDL17 and HDL18 particles in the TB cohort when compared to the Mihaleva cohort. Moreover, we found high CH and TG concentrations in CM, which is in clear contrast to the very low concentrations found in the Mihaleva cohort. Considering the continuous transition from CM to VLDL particles, it is evident, that also the TG and CH concentrations in the larger VLDL particles (VLDL03-VLDL05) were increased in the TB cohort. In comparison, the TG and CH concentrations in the VLDL06 and VLDL07 particles were similar in both cohorts. There were also no significant differences in the TG and CH concentrations in the LDL particles between the TB and Mihaleva cohorts. Only the CH concentrations in the LDL08 particles were lower in subjects of the TB cohort when compared to the Mihaleva cohort.

### Comparison of LP models between two study cohorts

Regarding the performances of the TB and Mihaleva PLS models (Figure S2), the TB model delivered in general better predictabilities for the VLDL-CH, LDL-TG, and LDL-CH subclasses, while the Mihaleva model resulted in higher Q^2^-values for the HDL-TG, HDL-CH, and VLDL-TG subclasses. Differences in TG and CH concentrations in these particles may partially explain the different performances. Most obviously, the TG and CH concentrations in the CMs were too low in the Mihaleva cohort to be predicted. Moreover, the poor performance of the HDL15-CH and HDL15-TG TB models may be attributed to the low CH and TG concentrations in the HDL15 particles.

**Figure 2:**
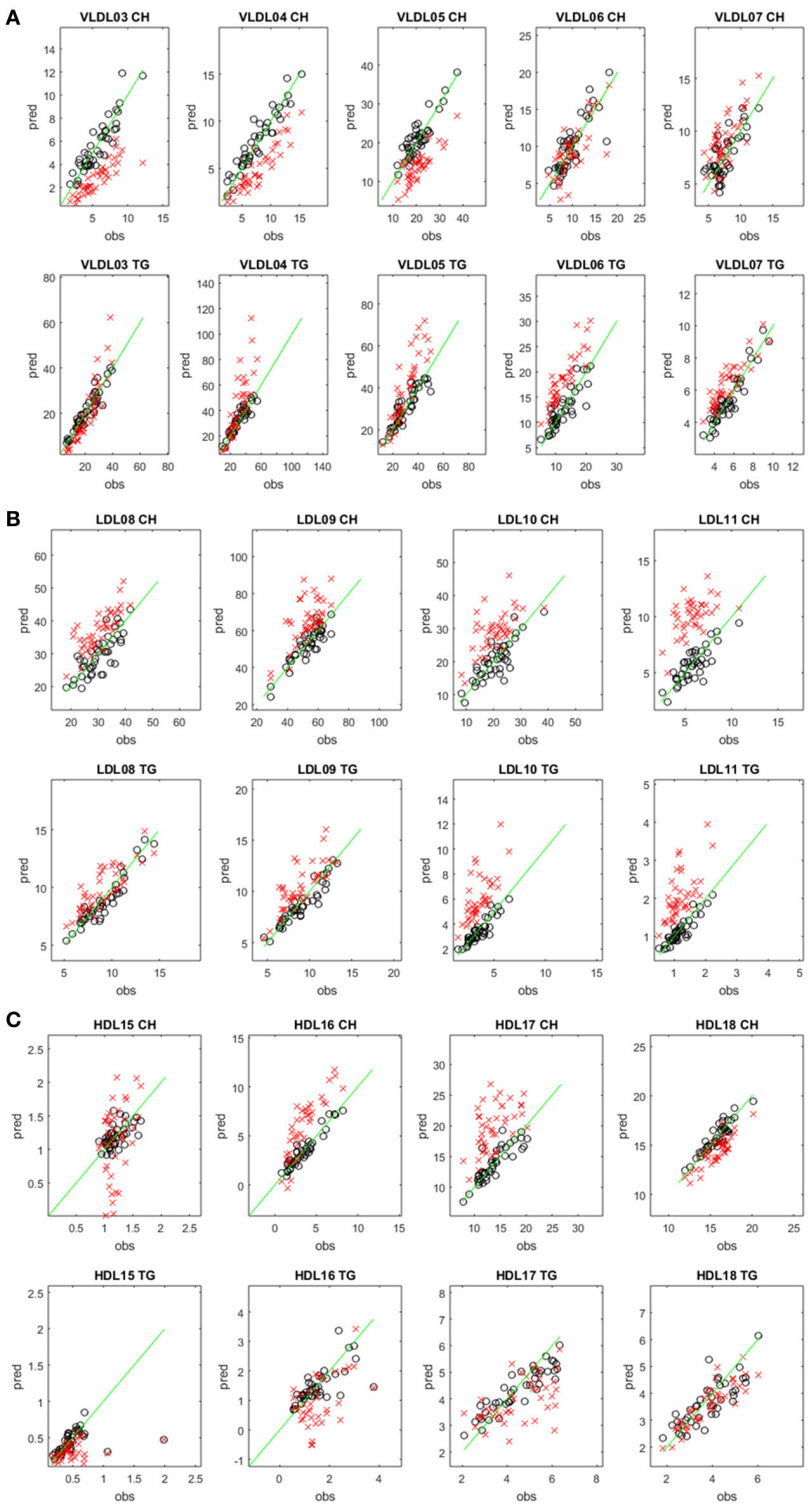
Comparison between predicted and measured CH and TG concentrations in different lipoprotein subclasses. Predicted (pred) versus measured (obs) CH (upper rows) and TG (lower rows) concentrations (in mg/dL) in the VLDL **(A)**, LDL **(B)**, and HDL **(C)** subclasses. The concentrations were predicted from the diffusion-edited ^1^H NMR spectra of the test set samples of the TB study using the TB model (black circles) and the Mihaleva model (red crosses), respectively. The measured concentrations were from the 42 test set samples of the TB study. The green line shows the ideal correlation between the predicted and measured concentrations.

We also predicted the TG and CH concentrations of the test set samples of the TB cohort using the Mihaleva model and compared these predictions with those of the TB model (Figure 2). Overall, we found good correlations between the observed and predicted concentrations from the Mihaleva model (Figure S3). However, we observed a bias for most LP subclasses. For example, the predicted CH concentrations of VLDL03, VLDL04, and VLDL05 were underestimated, while the predicted TG concentrations of the VLDL06, VLDL07 were overestimated. In comparison, the Mihaleva model performed reasonably well for VLDL06-CH, VLDL07-CH, VLDL03-TG, VLDL04-TG, and VLDL05-TG as long as the concentrations were low. High concentrations were still overestimated by most of these models. Regarding the LDL models, most TG and CH concentrations were overestimated from the Mihaleva model. The predictions of the TG and CH concentrations in the HDL particles delivered mixed outcomes. The predicted values of HDL18-TG from the Mihaleva model were comparable to the TB model, while the HDL15-CH concentrations were incorrectly predicted in most cases. In comparison, the predicted values for HDL16-CH, HDL17-CH were mostly overestimated, and the values for HDL16-CH, HDL15-TG, HDL16-TG and HDL17-TG were mostly underestimated.

### Effect of theobromine on CH and TG concentration in LP subclasses

We compared the effect of TB consumption to the placebo treatment on the TG and CH concentrations in 15 LP subclasses and in total VLDL, LDL, and HDL (Tables 3, 4). The TB consumption did not alter total HDL-CH. There were also no statistically significant changes in TG and CH in any of the HDL subclasses. Similarly, no changes in TG and CH concentrations were found in the CM and VLDL particles. We only observed significant reductions of CH in total LDL and in all LDL subclasses. CH was reduced by 5.24% in total LDL and by 3.64–6.79% in the different LDL subclasses. The strongest reductions were found in the LDL09 and LDL10 subclasses. The TG concentrations in the LDL particles did not change upon the TB treatment.

**Table 3:** The effect of theobromine (TB) and placebo treatments on serum cholesterol (CH) concentrations in total lipoproteins and 15 lipoprotein subclasses.

**LP class**	**Treatment**	**LSMeans [mM]^a^**	**Standard error [mM]^a^**	**Diff LSMeans TB vs. placebo [%]**	***p*-value^b^**
CM01-CH	placebo	0.076	0.008		
	TB	0.089	0.008	16.19	0.09
CM02-CH	placebo	0.047	0.004		
	TB	0.054	0.004	13.50	0.08
VLDL03-CH	placebo	0.148	0.008		
	TB	0.157	0.008	6.41	0.15
VLDL04-CH	placebo	0.213	0.010		
	TB	0.219	0.010	2.81	0.44
VLDL05-CH	placebo	0.573	0.011		
	TB	0.565	0.010	−1.53	0.32
VLDL06-CH	placebo	0.282	0.007		
	TB	0.277	0.007	−1.81	0.45
VLDL07-CH	placebo	0.221	0.006		
	TB	0.217	0.006	−1.79	0.32
LDL08-CH	placebo	0.823	0.018		
	TB	0.793	0.018	−3.64	**0.03**
LDL09-CH	placebo	1.424	0.038		
	TB	1.346	0.038	−5.48	**<0.0001**
LDL10-CH	placebo	0.571	0.016		
	TB	0.532	0.016	−6.79	**0.008**
LDL11-CH	placebo	0.159	0.005		
	TB	0.150	0.005	−5.68	**0.01**
HDL15-CH	placebo	0.034	0.001		
	TB	0.034	0.001	−0.05	0.97
HDL16-CH	placebo	0.106	0.006		
	TB	0.110	0.006	4.59	0.33
HDL17-CH	placebo	0.396	0.011		
	TB	0.401	0.011	1.37	0.41
HDL18-CH	placebo	0.421	0.005		
	TB	0.419	0.005	−0.47	0.61
VLDL_tot_-CH	placebo	1.421	0.033		
	TB	1.422	0.033	0.06	0.97
LDL_tot_-CH	placebo	3.017	0.081		
	TB	2.859	0.081	−5.24	**<0.0001**
HDL_tot_-CH	placebo	1.114	0.021		
	TB	1.126	0.021	1.04	0.35

a *Least Squares (LS)Means and Standard Errors were corrected for baseline, gender, age, body mass index, and two visits*.

b *p-value of Diff LSMeans TB vs. placebo; values in bold are statistically significant*.

**Table 4:** The effect of theobromine (TB) and placebo treatments on serum triglyceride (TG) concentrations in total lipoproteins and 15 lipoprotein subclasses.

**LP class**	**Treatment**	**LSMeans [mM]^a^**	**Standard error [mM]^a^**	**Diff LSMeans TB vs. placebo [%]**	***p*-value^b^**
CM01-TG	placebo	0.136	0.017		
	TB	0.161	0.016	18.43	0.11
CM02-TG	placebo	0.095	0.009		
	TB	0.106	0.009	11.21	0.17
VLDL03-TG	placebo	0.239	0.018		
	TB	0.255	0.018	6.41	0.25
VLDL04-TG	placebo	0.351	0.021		
	TB	0.361	0.021	2.73	0.50
VLDL05-TG	placebo	0.357	0.014		
	TB	0.356	0.014	−0.28	0.92
VLDL06-TG	placebo	0.150	0.004		
	TB	0.147	0.004	−1.46	0.54
VLDL07-TG	placebo	0.066	0.002		
	TB	0.066	0.002	−0.88	0.64
LDL08-TG	placebo	0.111	0.003		
	TB	0.109	0.003	−2.47	0.20
LDL09-TG	placebo	0.108	0.002		
	TB	0.105	0.002	−2.35	0.15
LDL10-TG	placebo	0.042	0.001		
	TB	0.042	0.001	−1.11	0.65
LDL11-TG	placebo	0.014	0.000		
	TB	0.014	0.000	0.29	0.92
HDL15-TG	placebo	0.005	0.000		
	TB	0.006	0.000	1.55	0.75
HDL16-TG	placebo	0.020	0.001		
	TB	0.021	0.001	2.49	0.65
HDL17-TG	placebo	0.056	0.002		
	TB	0.056	0.002	0.62	0.84
HDL18-TG	placebo	0.045	0.001		
	TB	0.045	0.001	0.33	0.91
VLDL_tot_-TG	placebo	1.169	0.057		
	TB	1.189	0.056	1.69	0.61
LDL_tot_-TG	placebo	0.282	0.006		
	TB	0.279	0.006	−1.22	0.46
HDL_tot_-TG	placebo	0.156	0.005		
	TB	0.156	0.005	0.46	0.86

a *Least Squares (LS)Means and Standard Errors were corrected for baseline, gender, age, body mass index, and two visits*.

b *p-value of Diff LSMeans TB vs. placebo; values in bold are statistically significant*.

## Discussion

The unique analytical advantages of NMR for LP profiling are the robustness, fast data acquisition and minimal sample preparation, which allow for automated analysis of thousands of serum samples (Soininen et al., 2015; van Duynhoven and Jacobs, 2016). Information on the LP subclass characteristics are usually extracted from the NMR spectra by line shape analysis or statistical regression analysis. The principle behind the statistical regression analysis is to first build a calibration model that correlates biochemical data (such as TG and CH concentrations) from LP subclasses to the NMR spectra of reference samples, and then to apply this model to predict the biochemical parameters from NMR spectra of other external test samples. In this way, samples from large studies can be analyzed in a time- and cost-effective manner. However, the statistical performance of the calibration model is crucial. This calibration model does not only define which LP properties and LP subclasses can be predicted, but also determines the error measures of the predicted values, which depend on the LP distribution of the reference population. Calibration models that are able to distinguish between different LP phenotypes must cover the LP distributions of all these phenotypes. Such models already exist for disease risk assessment and diagnostics (Vehtari et al., 2007). In contrast to these broad models, we have previously built a calibration model for a more narrow population to gain a higher accuracy in the predictions of TG and CH LP concentrations when studying nutritional effects (Mihaleva et al., 2014). Our Mihaleva cohort included apparently healthy men and women with borderline-high or high total cholesterol concentrations (5 and 8 mmol/L; 4.4–9.2 mmol/L; Mean±SE: 6.37 ± 0.05 mmol/L; Ras et al., 2014). Their total HDL-CH concentration ranged from 0.7–3.1 mmol/L Mean ± SE: 1.68 ± 0.02 mmol/L). In the current TB cohort, apparently healthy subjects with low total HDL-CH were recruited with concentrations ranging from 0.7 to 1.6 mmol/L (Mean ± SE: 1.08 ± 0.03 mmol/L) and thus being on average lower than in the Mihaleva cohort. Nevertheless, the Mihaleva calibration model was expected to be able to correctly predict the LP distribution from the TB cohort, because the Mihaleva model also covered the low HDL-CH concentrations (Figure S4). However, we found significant differences in the predicted concentrations of the independent TB test set which was applied to both the TB calibration model and the Mihaleva calibration model. Although, the predicted TG and CH concentrations correlated reasonably well with the actual concentrations when the test set of the TB cohort was applied to the Mihaleva model, the concentrations were over- or underestimated for the majority of LP subclasses. This bias may be explained by differences in the LP distributions of the TB and Mihaleva cohorts as shown in Figure 1. Most obviously, TG and CH in CM were detected in the TB cohort and not in the Mihaleva cohort. This means that CM signals were present in the ^1^H NMR spectra of the TB cohort and not in those of the Mihaleva cohort, which were not taken into account in the Mihaleva model. Thus, when applying the TB test set to the Mihaleva model, the Mihaleva model had to cope with the additional CM signals, which positively or negatively contributed to the predicted concentrations depending on the PLS coefficients. Therefore, even when the TG and CH concentrations in the test samples were within the concentration range of the Mihaleva model, the Mihaleva model might predict over- or underestimated values. For example, the TG and CH concentrations in all LDL particles were mostly overestimated, although the Mihaleva model covered the concentration ranges of the TB test set.

In addition, the concentration ranges in some LP subclasses in the TB test set were outside the ranges of the Mihaleva model. For example, the CH concentrations in the VLDL03-VLDL05 and HDL17 particles were higher or lower in TB test samples, when compared to the Mihaleva model. This may have led to overestimated HDL-CH and underestimated VLDL-CH predictions, because the model has forced the out-of-range concentrations into the model. Moreover, small differences in sample collection and NMR data acquisition may have also contributed to the prediction errors, even though the sample collection and NMR data acquisition protocols of both studies were identical. A ring test has recently shown, that the NMR LP profiles acquired on three NMR instruments from different labs were extremely reproducible, leading to prediction errors below 5% (Monsonis Centelles et al., 2017). From this cross–study comparison we conclude that LP prediction models should not be blindly applied to different studies without prior sanity checks.

In the TB study, subjects with low baseline HDL-CH concentrations were recruited. We found low CH concentrations in the larger HDL particles (HDL15-17) but not in the smallest HDL particle (HDL18) when comparing the TB and Mihaleva cohorts. In addition, having low HDL-CH was accompanied with high TG and CH concentrations in large VLDL and CM particles. Physiologically, TG carried in VLDL and LDL is exchanged as CH ester carried in HDL. This process is facilitated by CETP. TG rich VLDL may enhance the movement of TG from VLDL to HDL for exchange of CH esters, which may provide an explanation why the lower HDL-CH concentrations were associated with high TG in VLDL in the TB cohort. In addition, the subjects were mostly overweight or obese (BMI: 29 ± 3 kg/m^2^). It is known that obese subjects are more likely to be insulin resistant (Maldonado-Ruiz et al., 2017). Impaired hepatic insulin signaling is related to increased TG-rich VLDL production (Santamarina-Fojo et al., 1998) which is typically associated with reduced HDL-CH and low or non-elevated LDL-CH. High TG and CH concentrations in large VLDL/CM as well as low TG and CH concentrations in large HDL particles as observed in subjects of the TB study may reflect an unfavorable metabolic condition since large VLDL particles have been related to increased risk of atherosclerosis (Rizzo et al., 2008; Wurtz et al., 2011) and CVD (Arsenault et al., 2009). Interestingly, a recent study has characterized several LP profiles among metabolically healthy and unhealthy individuals classified as obese and non-obese (Phillips and Perry, 2015). In this study, metabolically unhealthy individuals had high numbers of large VLDL and small LDL particles and low numbers of large LDL and large HDL particles, as opposed to metabolically healthy individuals. Interestingly, the (un)favorable LP phenotypes were not dependent on BMI and the definition of metabolic health.

In this study, TB induced a modest, but significant reduction in LDL-CH, which is in agreement with other short-term intervention studies on cocoa/dark chocolate products (Tokede et al., 2011). CH reductions were found in all LDL subclasses ranging from 3.64% in LDL08 to 6.79% in LDL09. This suggests that TB did not specifically target one specific LDL subclass such as the small dense LDL or the larger less dense LDL, the former being associated with higher risk of coronary heart disease (Berneis and Krauss, 2002). In contrast to LDL, TB did not alter the CH concentrations in total HDL and in any of the HDL subclasses. Reported effects of TB on plasma/serum HDL-CH concentration are conflicting. Several meta-analyses (Jia et al., 2010; Shrime et al., 2011; Tokede et al., 2011) of studies with cocoa (products) (including cocoa (products) rich in TB) did not conclude a HDL-CH increasing effect. A recent study did not show an increase in HDL-CH in overweight adults upon 4-week consumption of cocoa containing 476 mg TB (West et al., 2014). However, other studies in healthy subjects and those at high risk of CVD showed that TB dose-dependently (150–1,000 mg/d) increased HDL-CH by 2.9–18% within 4-12 weeks (Wan et al., 2001; Baba et al., 2007; Taubert et al., 2007; Monagas et al., 2009; Khan et al., 2012; Neufingerl et al., 2013). Reasons for these conflicting data have not conclusively emerged yet. It is unclear whether for instance the LP phenotype at baseline plays a role. Clearly, the subjects in the present TB study had a LP phenotype (low HDL-CH, high CM-TG, high VLDL-TG) that differed from subjects in the Mihaleva study, and from those in a previous study, in which TB was shown to increase HDL-CH (Neufingerl et al., 2013). Differences in serum LP profiles may reflect variations in lipid metabolism that may affect the efficacy of TB. Further studies are required to find out whether impaired HDL functionality impacts TB efficacy. Moreover, most studies have observed an HDL-CH increase using cocoa (products) (Wan et al., 2001; Baba et al., 2007; Taubert et al., 2007; Monagas et al., 2009; Khan et al., 2012), with the exception of the study by Neufingerl et al. (2013), who found this increase also upon TB consumption. Thus, it may be worthwhile to further investigate, whether TB alone or together with other components in cocoa such as polyphenols will lead to HDL-CH increasing effects. However, we suggest to perform these studies in cohorts with normal HDL-CH.

In summary, LP prediction from diffusion-edited ^1^H NMR spectra is a useful approach for determining LP distributions, provided that the PLS model is properly set up according to the LP characteristics of the target cohort. Using this approach, the LP phenotype of this study cohort was characterized by low CH in HDL subclasses and high TG in VLDL and CM subclasses. In such phenotypic subjects, TB failed to show an increase in HDL-CH, while it reduced CH in all the LDL subclasses. These findings may provide an explanation of the conflicting literature data regarding an HDL-CH increasing effect of TB.

## Author contributions

DJ contributed to the LP analysis, performed the statistical analysis and wrote the manuscript; LS, RM, and JP conceived and performed the intervention study; NdR acquired the NMR spectra, VM predicted the LP distributions; JvD contributed to the LP analysis; YL and ET contributed to study design and the formulation of TB drink for the clinical study; YL contributed to the manuscript preparation.

### Conflict of interest statement

DJ, YL, Nd, ET, Jv, and VM are employed by Unilever R&D. Unilever markets products with a heart health benefit. The other authors declare that the research was conducted in the absence of any commercial or financial relationships that could be construed as a potential conflict of interest.

## Abbreviations

CHD: coronary heart disease
CH: cholesterol
CM: chylomicrons
HDL: high-density lipoprotein
LDL: low-density lipoprotein
LP: lipoproteins
TG: triglycerides
VLDL: very high-density lipoprotein.
