# Supplementary material for: Effect of Theobromine Consumption on Serum Lipoprotein Profiles in Apparently Healthy Humans with Low HDL-Cholesterol Concentrations
Source: Front Mol Biosci. 2017 Aug 24;4:59. doi: 10.3389/fmolb.2017.00059 (PMC5609577; doi:10.3389/fmolb.2017.00059)
Supplement: Supplementary file 1 [file Presentation1.pptx]

## Slide 1
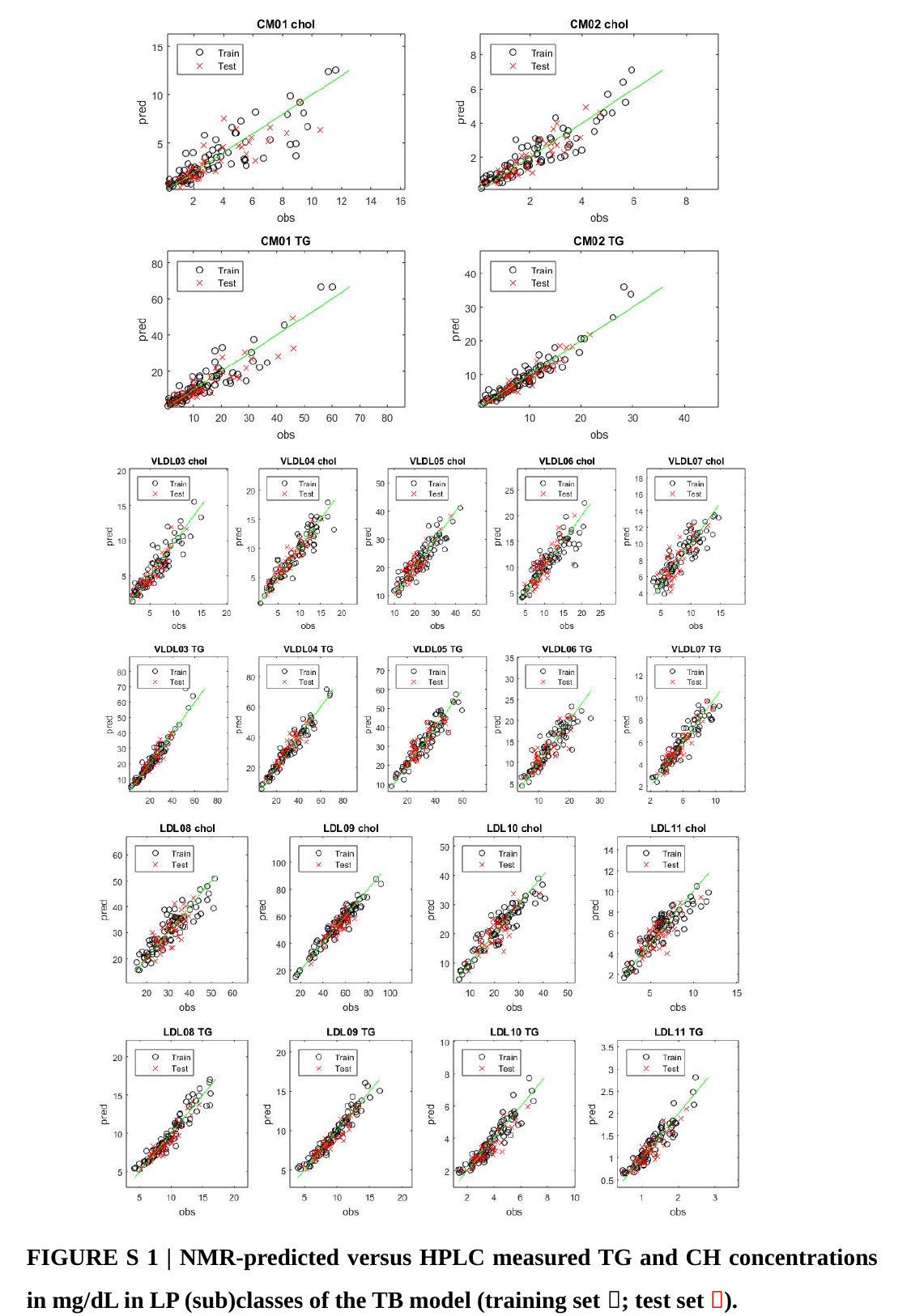

Figure S 1 | NMR-predicted versus HPLC measured TG and CH concentrations in mg/dL in LP (sub)classes of the TB model (training set ; test set ).

## Slide 2
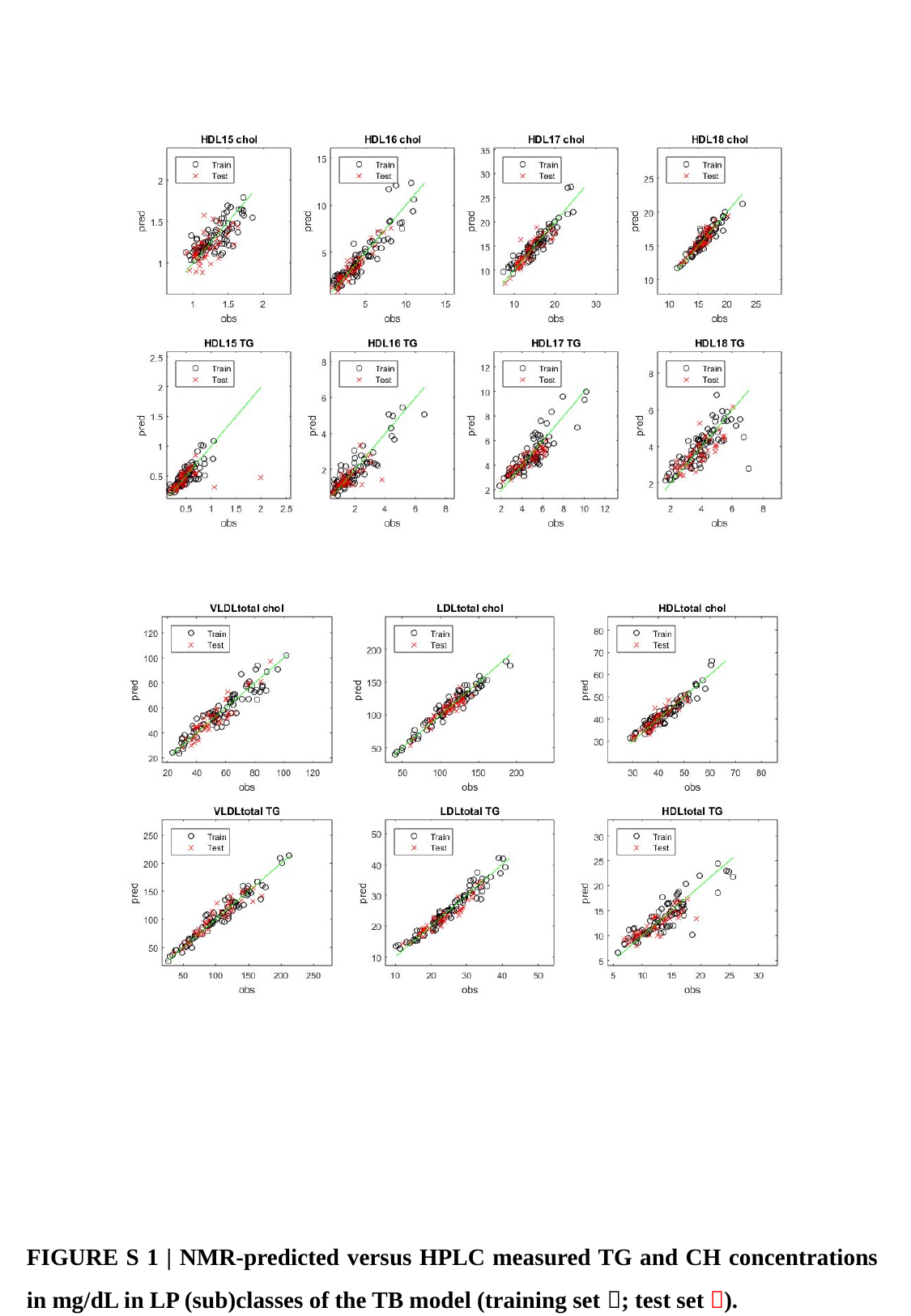

Figure S 1 | NMR-predicted versus HPLC measured TG and CH concentrations in mg/dL in LP (sub)classes of the TB model (training set ; test set ).

## Slide 3
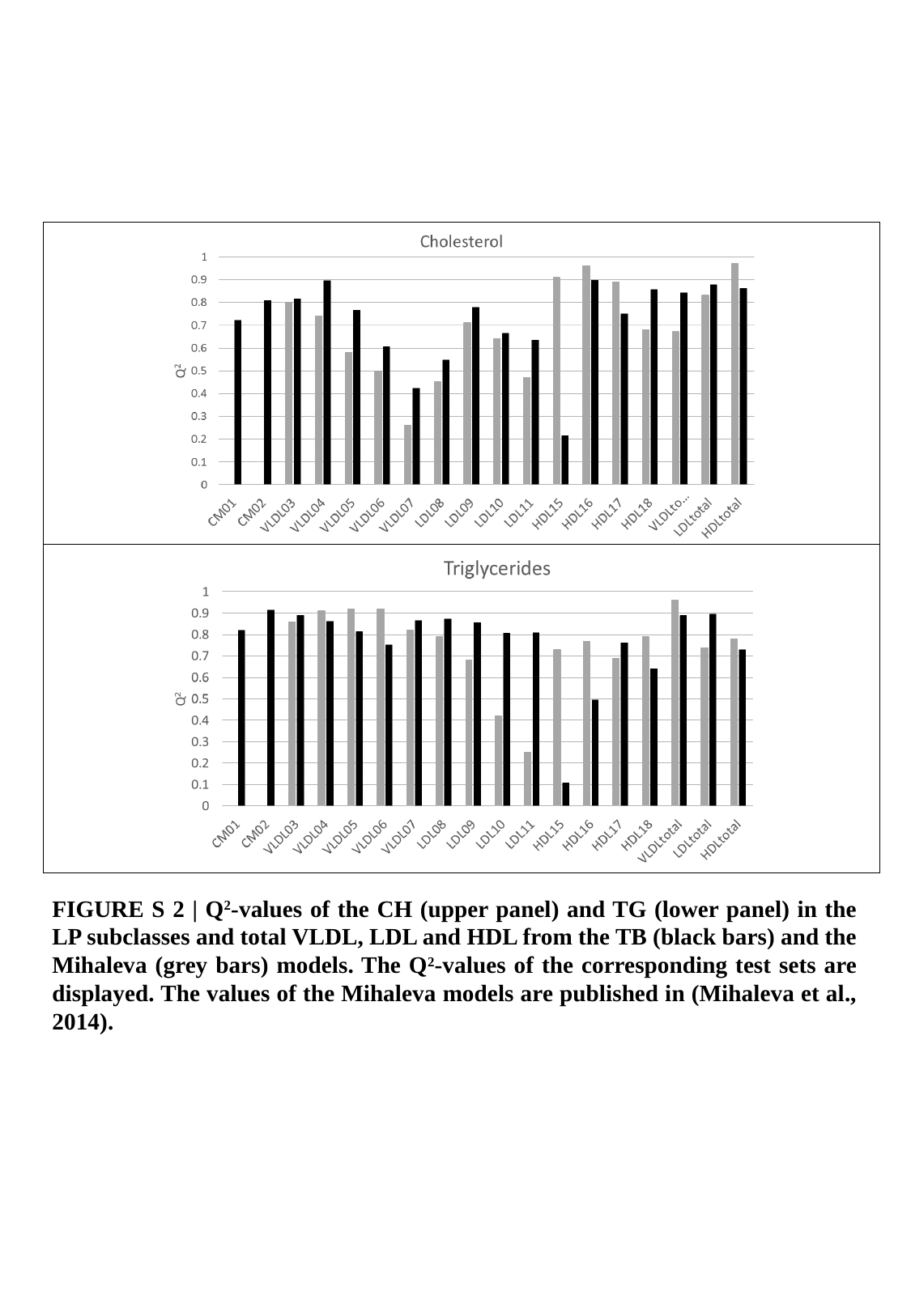

FIGURE S 2 | Q2-values of the CH (upper panel) and TG (lower panel) in the LP subclasses and total VLDL, LDL and HDL from the TB (black bars) and the Mihaleva (grey bars) models. The Q2-values of the corresponding test sets are displayed. The values of the Mihaleva models are published in (Mihaleva et al., 2014).

## Slide 4
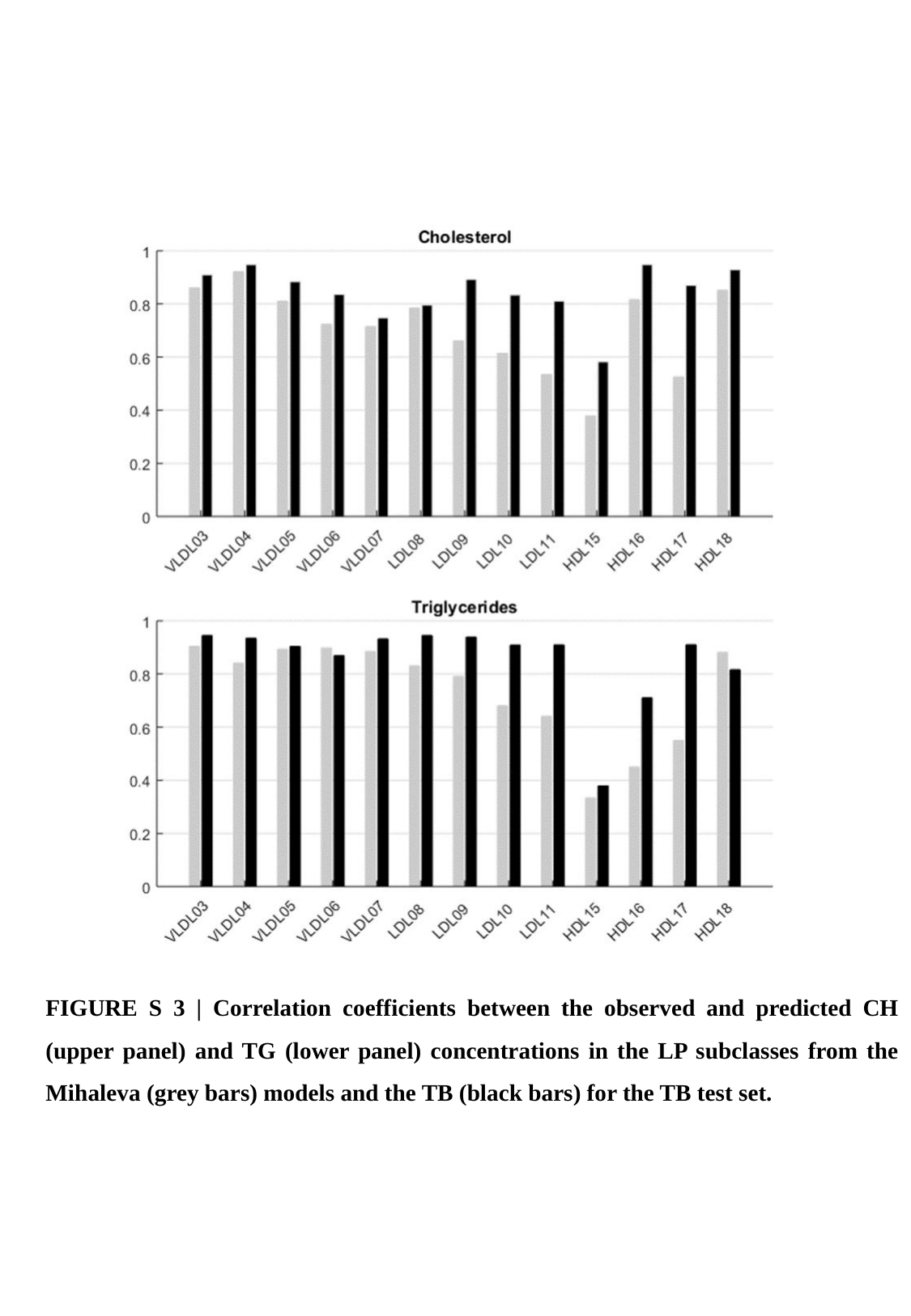

Figure S 3 | Correlation coefficients between the observed and predicted CH (upper panel) and TG (lower panel) concentrations in the LP subclasses from the Mihaleva (grey bars) models and the TB (black bars) for the TB test set.

## Slide 5
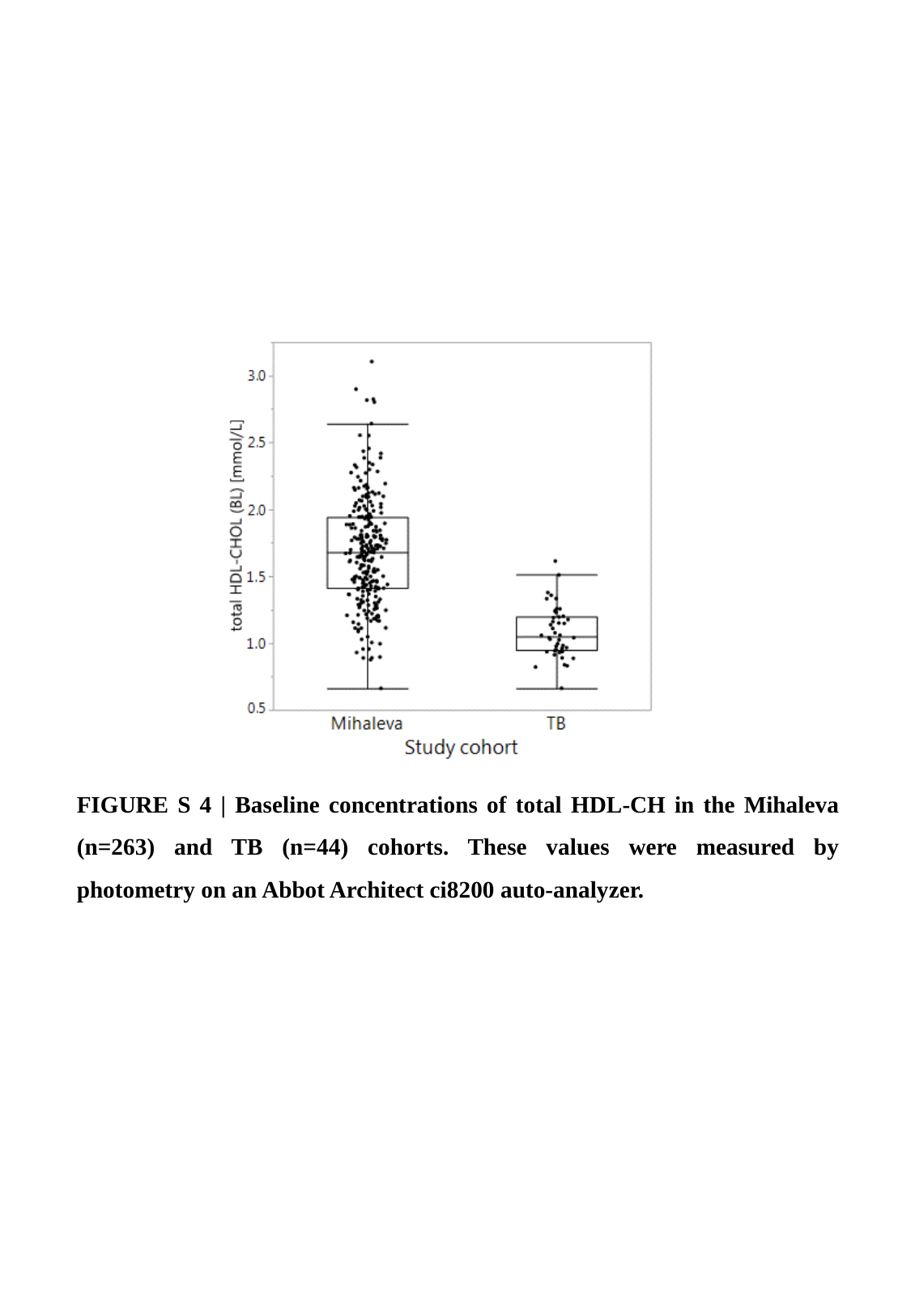

Figure S 4 | Baseline concentrations of total HDL-CH in the Mihaleva (n=263) and TB (n=44) cohorts. These values were measured by photometry on an Abbot Architect ci8200 auto-analyzer.
